# How molecular imaging will enable robotic precision surgery: The role of artificial intelligence, augmented reality, and navigation

**DOI:** 10.1007/s00259-021-05445-6

**Published:** 2021-06-29

**Authors:** Thomas Wendler, Fijs W. B. van Leeuwen, Nassir Navab, Matthias N. van Oosterom

**Affiliations:** 1 Chair for Computer Aided Medical Procedures and Augmented Reality, Technische Universität München, Boltzmannstr. 3, 85748 Garching bei München, Germany; 2 Department of Radiology, Interventional Molecular Imaging Laboratory, Leiden University Medical Center, Leiden, The Netherlands; 3 Department of Urology, The Netherlands Cancer Institute - Antonie van Leeuwenhoek Hospital, Amsterdam, The Netherlands; 4 Orsi Academy, Melle, Belgium; 5 Chair for Computer Aided Medical Procedures Laboratory for Computational Sensing + Robotics, Johns-Hopkins University, Baltimore, MD USA

**Keywords:** Robotic surgery, Molecular imaging, Image-guided surgery, Precision surgery, Artificial intelligence, Augmented reality

## Abstract

Molecular imaging is one of the pillars of precision surgery. Its applications range from early diagnostics to therapy planning, execution, and the accurate assessment of outcomes. In particular, molecular imaging solutions are in high demand in minimally invasive surgical strategies, such as the substantially increasing field of robotic surgery. This review aims at connecting the molecular imaging and nuclear medicine community to the rapidly expanding armory of surgical medical devices. Such devices entail technologies ranging from artificial intelligence and computer-aided visualization technologies (software) to innovative molecular imaging modalities and surgical navigation (hardware). We discuss technologies based on their role at different steps of the surgical workflow, i.e., from surgical decision and planning, over to target localization and excision guidance, all the way to (back table) surgical verification. This provides a glimpse of how innovations from the technology fields can realize an exciting future for the molecular imaging and surgery communities.

To optimize patient outcomes and accommodate public demand, surgical approaches are becoming more and more tailored to the individual’s needs. This personalization is pursued to the extent that precision surgery is one of the leading trends in current medicine [1]. Not only is there a drive towards increasing the resection accuracy, but there also is an increasing focus on balancing cure and side effects, requiring precise (intraoperative) target definition and control. These trends have driven the growth of minimally invasive surgery, particularly the implementation of robotic surgery and various image-guided surgery technologies.

In the past decades, the availability of robotic telemanipulators or robotic master-slave systems has facilitated robot-assisted surgery. In particular, the da Vinci platform (Intuitive Surgical, Sunnyvale (CA), USA), as the prime example, has become the new standard for the management of prostatic cancer (i.e., prostatectomy and lymphatic dissections) [2]. An indication where patients are increasingly being staged based on molecular imaging (i.e., PSMA PET [3]). The first generation of telemanipulator systems has sparked the dissemination of robotic surgery to other indications, e.g., lymphatic dissections [4], partial nephrectomy [5], hysterectomy [6], pulmonary and esophageal surgery [7], hepatobiliary surgery [8], colorectal cancers [9], and even head and neck surgery [10]. With the routine inclusion of a fluorescence laparoscope (since the da Vinci Si model) and the TilePro function that facilitates the multi-input display of preoperative scans (e.g., PET or SPECT) and intraoperative imaging directly within the surgical console, robotic surgery has well geared up. These upgrades facilitate efficient integration of all the data streams associated with the use of image-guided surgery (Fig. 1).

**Fig. 1:**
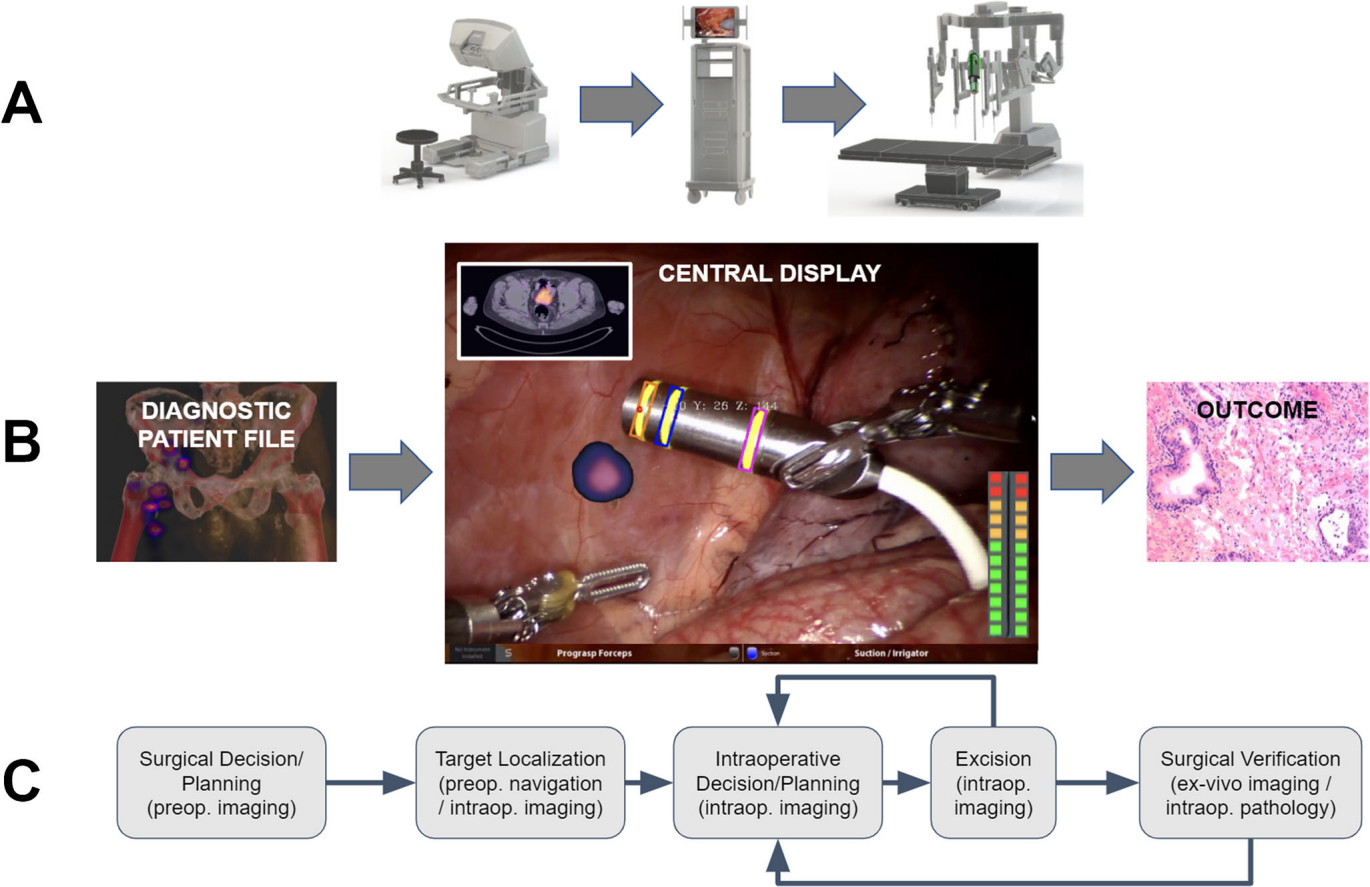
**A** Components in a robotic telemanipulator system. The surgeon operates the robot from a console that connects to the robotic arms over a central data processing unit where also the video signal of the laparoscope is processed. **B** Molecular images, like PET, SPECT, or scintigraphy, and so-called metadata of the patient are fed to the data processing unit. There, intraoperative information is merged and shown in a single central display. There, augmented reality (AR) image overlays can be shown with the instruments and signals from the surgery (theoretical possibilities indicated). As a result of the procedure, the diseased tissue is removed. This results in an outcome, e.g., the resection borders’ status. **C** A molecular imaging-enhanced robotic surgery can be abstracted as surgical decision/planning, target localization, intraoperative decision/planning, excision, and surgical verification, all of which are interconnected

Beyond the da Vinci system, more robotics platforms are now making their way into clinical care. The Versius (CMR Surgical, Cambridge, UK) and the Senhance surgical system (ransEnterix, Morrisville (NC), USA) follow a similar approach to Intuitive Surgical with a versatile model applicable to several anatomies. However, other surgical robots have entered niche “organ” markets like neurosurgery and orthopedics, e.g., the ROSA brain and knee robots (Zimmer Biomet, Warsaw (IN), USA), the Mazor X spine robot (Medtronic, Dublin, Ireland), the Mako knee surgery assistant (Stryker, Kalamazoo (MI), USA) and the Navio knee robot (Smith & Nephew, Watford, UK). The field also extends into more complex indications like natural orifice total endoscopic surgery, e.g., the Flex robotic system (Medrobotics, Raynham (MA), USA), peripheral lung biopsies, e.g., the Ion (Intuitive Surgical, Sunnyvale (CA), USA), or the Monarch system (Auris Health, Redwood City (CA), USA), or catheter guidance, e.g., the CorPath GRX (Corindus, Waltham (MA), USA) or the Vdrive system (Stereotaxis, St. Louis (MO), USA). The expansion of robotics will go further in the direction of microrobotics, as needed in eye or brain surgery [11, 12].

Image guidance (and progressively molecular image guidance) is probably one of the most critical levers in realizing precision surgery. Three aspects are strongly contributing to its evolution. First, advances made in radiology and nuclear medicine have made it possible to identify diseased tissue early and with superior accuracy and molecular precision [13]. In particular, molecular imaging plays a significant role in this improvement; Molecular imaging refers to the in vivo imaging of molecule concentrations based on the presence of an endogenous molecule or the injection of an exogenous imaging agent (i.e., tracer Table 1). In vivo detection succeeds utilizing a radioactive, fluorescent, or magnetic label (exogenous tracers, Table 1) or by an optical or electrical signature of the target molecule in a non-contrasted approach [14, 15]. Second, the rise of interventional molecular imaging strategies, in the form of tracers and surgical molecular imaging modalities, increasingly allows surgeons to target and resect tissues based on molecular features [16]. This can be, for example, using radio-guided surgery [17, 18] or fluorescence-guided surgery [19]. Third, in the surgical arena, significant strides have been made concerning image guidance modalities (hardware) and visualization concepts (software) [20].

**Table 1:** Robotic indications where tracer-based molecular imaging has already clinically demonstrated value in surgical planning, intraoperative guidance, and postoperative control

Indication	Tracer	Preop. imaging	Intraop./ex vivo detection	Reference
Lymphatics				
(Sentinel) Lymph node biopsy	^99*m*^*Tc*-radiocolloids	Sz, SPECT/CT	DROP-IN *γ* Counting, ioSz, fhSPECT	[21]
	ICG-^99*m*^*Tc*-HSA nanocolloid	Sz, SPECT/CT	DROP-IN *γ* Counting, ioSz, fhSPECT, FluoLap	[22]
	ICG	-	FluoLap	[23]
	Fluorescein	-	FluoLap	[24]
	Methlyene blue	-	FluoLap	[25]
High blood perfusion				
Tumors (e.g., liver, adrenal glands)	ICG	-	FluoLap	[26]
Vessels (e.g., ureters, anastomosis, vasculature)	ICG	-	FluoLap	[27]
Receptor-targeted				
Prostate cancer	^99*m*^*Tc*-PSMA I&S	Sz, SPECT/CT	DROP-IN *γ* Counting, ioSz, fhSPECT	[28]
	^68^*Ga*-PSMA-914	PET/CT, PET/MR	DROP-IN β Counting (experimental), FluoLap, Cerenkov Imaging	[29]
Clear cell renal cell carcinoma (ccRCC)	^111^*In*-DOTA-girentuximab IRDye800	Sz, SPECT/CT	DROP-IN *γ* Counting, ioSz, fhSPECT, FluoLap	[30]
	OTL38	-	FluoLap	[31]
Pulmonary nodules	OTL38	-	FluoLap	[32]

*io*, intraoperative; *Sz*, scintigraphy; *fhSPECT*, freehand SPECT; *FluoLap*, fluorescence laparoscopy

Since the robotic setting is somewhat different from a traditional operating room setting, many current image guidance modalities have to be specifically adapted. However, since most robotic platforms harbor a plural of highly maneuverable instruments, this does open up many new advantages that could lead to a better performance of the image guidance modalities themselves [33]. Furthermore, since the surgeon is operating behind a video console or “central display” (see Fig. 1A and B), the robotic platform seems to be an ideal system to integrate new developments in display technology, directly visualizing all kinds of patient information (e.g., [34]). Such patient datasets could entail pre- or intraoperative imaging, surgical planning, navigation, patient monitoring, and (post-resection) lesion confirmation. With the high amounts of computational power available with current-day technology, such datasets’ formation is increasingly assisted with artificial intelligence (AI) methods, including machine learning, and in particular deep neural networks. Thus, the establishment of robotic surgery requires the necessary adjustments but provides many exciting opportunities for molecular image-guided surgery.

This review aims at connecting the molecular imaging and nuclear medicine community, which provides a wide range of radiotracers for image-guided surgery, to current hardware and software developments. This entails technologies ranging from artificial intelligence and advanced visualization to robot-tailored imaging modalities. We will review works based on the robotic surgery workflow (see Fig. 1). To provide insight into the direction that the field is moving in, we have mainly focused on technological possibilities that are becoming available or are expected to provide future impact for robot-assisted surgery.

To guarantee a thorough literature review, here we employed two approaches of research. First, we performed an exhaustive search on the highest impact *Molecular Imaging* and *Medical Robotics* journals to identify the most relevant articles and reviews of the field over the last 10 years. Secondly, a systematic and comprehensive keyword-based search was conducted on *molecular imaging in a minimally invasive or robotic setup* in PubMed,^1^ Google Scholar,^2^ and Semantic Scholar^3^ combining the keywords Molecular Imaging, Nuclear Imaging, Gamma Imaging, Beta Imaging, Fluorescence Imaging with the terms Image-guided surgery, Laparoscopic surgery, Minimally-invasive surgery, and Robotic surgery. All titles and abstracts were scanned manually for relevance, deleting duplicates and selecting the ones that best depicted the techniques searched in the view of the authors, prioritizing in-human trials while only including the most promising non-human trials.

## Preoperative planning and navigation

Preoperative molecular images, like SPECT and PET, nowadays almost always in combination with CT or MRI, are the starting point of many robotic surgery procedures. They serve as means to select patients, plan entry paths, trocar placement, and then even, in some cases, guide the surgeon grossly to the area of the target.

### Preoperative molecular imaging for surgical selection, planning, and prediction

The selection of patients for surgery has been a task of the surgeon or a multidisciplinary group of experts. Such a process often involves considering the patient metadata (health state, age, occupation, family history), available know-how, and infrastructure, as well as imaging material to assess if surgery is the most recommendable option. The surgical risk can also be estimated by looking at organs at risk close to the surgical site and potential access paths.

Routine lymphatic mapping has made treating surgeons aware of the role that preoperative imaging plays in image guidance. Moreover, they have shown that the type of preoperative imaging defines the value created in the OR [35]. For example, complementing 2D scintigraphic images with 3D SPECT/CT increases the number of identified sentinel nodes. Equally important, the SPECT-registered (low dose) CT-based anatomical image (also in 3D) helps provide anatomical context, visualize specific surgical landmarks, and locate organs at risk. This allows surgeons to plan their surgical procedure by pinpointing the area of interest more accurately and make a risk estimation concerning the induction of damage to vital structures.

#### Artificial intelligence for segmentation

**Fig. 2:**
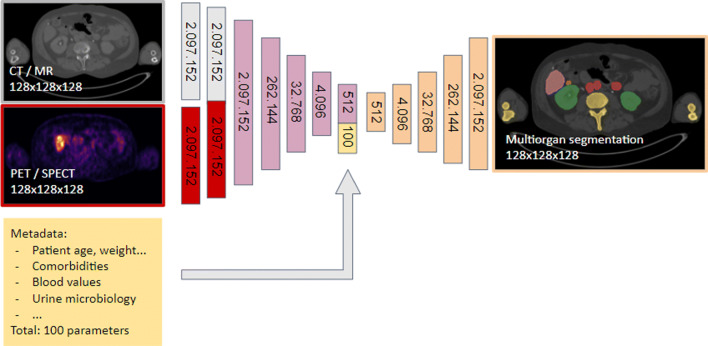
Example of a convolutional deep neural network (CNN), a standard AI algorithm. CNNs can be applied for multiorgan image segmentation using molecular imaging and metadata. Here, the CNN reads anatomical images (CT or MR, gray) and molecular images (PET/SPECT/scintigraphy, red). After initial processing, it concatenates their features (pink). The network then reduces the input’s dimensionality (i.e., brings the images from a size of, e.g., 128×128×128 = 2,097,152 to only values 512 representing both of them). These 512 parameters get further concatenated with the 100 metadata parameters (yellow) fed into the network’s bottleneck. The 512 + 100 = 612 parameters contain a compressed high-level representation of the image and patient information. Based on the image and metadata, the network then solves the target tasks (here organ segmentation, orange) while increasing dimensionality (i.e., upscaling from 612 parameters back to 128×128×128 = 2,097,152)

In the whole preoperative route of patient selection, surgical planning, and prediction, AI methods are starting to play an increasingly relevant role, assisting the physicians in delivering their best performance. So far, such AI methods have mostly focussed on the aspect of surgical planning, providing an automatic segmentation of tumors and organs at risk using both patient imaging and metadata. For such segmentation models, deep learning methods have predominantly taken over, often based on convolutional neural networks (CNNs). For image processing, such CNNs have been shown to overperform humans in classification and detection methods (e.g., [36, 37]). They are increasingly being used for tasks like multi-organ segmentation [38]. When dealing with imaging data, a CNN consists of stacked nonlinear convolutional layers that can extract features of high-level (shapes, content) and low-level (details, texture) from images. The network architectures reduce dimensionality first to extract high-level features in a so-called bottleneck representation of the image (see Fig. 2). From there, these features are further processed, where so-called skip connections contribute low-level information from early layers. In the end, the final layers convert the features into probabilities, for example, in the scenario of organ segmentation. AI organ segmentation can even be further optimized by feeding the neural network with relevant patient metadata, such as patient age, weight, clinical parameters, and previous treatment strategies (e.g., [39]).

To indicate organs at risk, such automatic multi-organ segmentations have been successfully tackled using CT scans [40–42] or MR scans [43], even in case they were cropped [44]. The higher the accuracy of these segmentation models (often expressed as a higher “Dice score”), the lower the need for an expert physician to correct the automatic segmentation. Interestingly, combining such anatomy-based segmentations with molecular imaging (i.e., PET or SPECT) can further improve the AI results. A network having both inputs can learn the physiological uptake of different organs and enhance the segmentation (e.g., improved definition of (metastatic) tumor nodules). First approaches that use both anatomical and molecular images to enhance segmentation have been undertaken with PET/CT in automatic tumor segmentation in lung cancer [45–47], head and neck tumors [48, 49], and non-Hodgkin lymphoma [50]. Here, a combination of the tumor detection task and the multi-organ segmentation can be advantageous as this mimics both nuclear medicine physicians and radiologists [51].

#### Artificial intelligence for outcome prediction

Next to assistance in surgical planning, AI methods such as deep learning might improve initial patient selection and even enable predicting treatment outcomes. The latter could entail the detection of abnormal anatomy during surgery, the occurrence of side effects, the duration of the procedure, or the length of the postoperative stay. Initial works have been published for the treatment of esophageal [52] and oropharyngeal cancer [53] using PET images as input. Despite the prediction being here the response to chemotherapy or radiotherapy, technically, the same approach can be used to train a system for surgical prediction by changing the training dataset to include surgical outcome information.

### Preoperative molecular imaging as intraoperative roadmaps for target localization

Preoperative imaging techniques are used routinely to create detailed roadmaps, based on which a surgeon can plan and execute its resection. Initially, surgeons study the diagnostic images together with their nuclear medicine colleagues. Based on these discussions, the surgeon would then create a mental roadmap of the procedure. While expert surgeons are surprisingly effective in doing such translations, this approach’s failure rate is relatively high (e.g., [54–57]). This has been the motivation behind initiatives that physically bring preoperative imaging information into the surgical theatre. We do not refer to the old-fashioned printout of, e.g., a 2D scintigraphic image, but rather the use of fully annotated digital images displayed on a dedicated dashboard in the OR. Such displays have been used since the beginning of medical imaging and, mainly, since PACS’s introduction in the 1990s [58]. The increasing complexity of 3D scans such as SPECT/CT or PET/CT has driven a growth in the use of such display options. The TilePro extension of the da Vinci surgical console now even makes it possible to directly input preoperative images (or other datasets) as windows in the surgeon’s display [59, 60]. Despite the availability of such displays, it remains challenging to translate such preoperative imaging information to successful surgical execution. This happens since the patient is drastically repositioned, covered by the surgical robot, insufflated, and visualized through the robotic laparoscope’s vision.

**Fig. 3:**
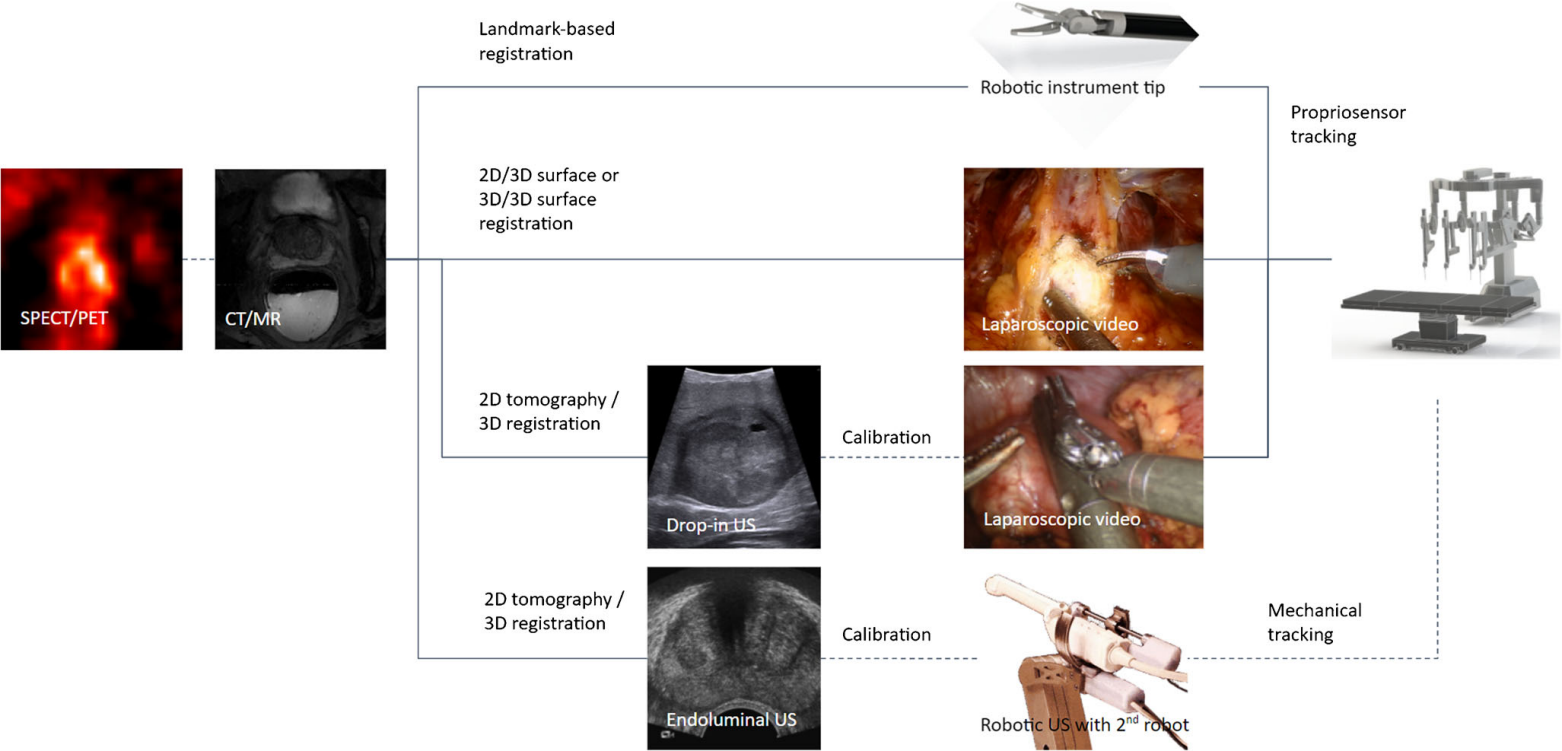
Possible registration options to bring molecular images (SPECT/PET or MRI) over anatomical images (CT/MR) to the robot’s coordinate system

The direct integration of the preoperative images into the surgeon’s laparoscopic view is a promising development field. This can be enabled with either an augmented reality (AR) overlay on the laparoscopic video feed or a virtual reality (VR) visualization. Both allow navigating towards tissue targets as marked in the preoperative images [61]. For these approaches to work, it is instrumental that the preoperative images are geometrically registered to the patient’s position and orientation (pose) in the operating room. In robotic surgery, such registration relies on tracking solutions, such as optical or mechanical tracking systems calibrated to the robot, or the robotic arms’ propriosensory information, to define the patient’s relative pose, laparoscopic instruments, and camera (Fig. 3). Several registration approaches can be followed to bring the preoperative images into the tracking system’s coordinate system.

#### Landmark-based registration

This is the most intuitive and straightforward option already proposed at the end of the 1990s [62]. First, particular landmarks are selected on the preoperative images and subsequently tipped with a calibrated tracked instrument in situ. The resulting point correspondences between preoperative images and tracking coordinates of the anatomical landmarks can be processed to yield the desired registration. The more anatomic landmarks used, the better the quality of registration. In practical terms, this is challenging and, unfortunately, often results in poor registrations. On the other hand, in robotic surgery, the robotic instruments are tracked using the propriosensory and can be used to tip the landmarks making an external tracking system unnecessary.

#### Laparoscopic video-based registration

Several groups have proposed methods for registering the laparoscopic video with renderings of the preoperatively acquired images [63, 64], either using a 2D or 3D laparoscope. Such approaches rely on the fact that preoperative CT or MRI depicts the anatomy at the time of surgery reasonably well (i.e., no significant resections or strong tissue deformations have taken place). Besides, the lack of clearly distinguishable landmarks in the laparoscopic images makes this registration approach challenging. However, since the robot’s propriosensors track the laparoscope’s pose, no additional tracking hardware is required here. Also, with the continuous development of AI-supported surgical scene recognition, it is expected that there is a lot to be won in the accuracy of this method (e.g., [65, 66]).

#### Intraoperative ultrasound-based registration

While registering organ surfaces to preoperative imaging is feasible using the laparoscopic video feed, deep-lying structures may not be properly registered. In a robotic setup, this could be achieved with a positionally tracked ultrasound (US) probe. More specifically, the probe can be either endoluminal ultrasound (i.e., transrectal or transvaginal ultrasound, tracked via mechanics or a second robot [67]) or DROP-IN ultrasound (tracked via laparoscopic-video [68]). The most important advantage is the possibility of using in-depth information to improve registration results. However, it is limited to specific applications and organs where US is feasible (such as the prostate, kidney, liver).

From the methods mentioned above, we believe that evolutions of laparoscopic video-based registration, and to a lesser extent, ultrasound registration, combined with mechanical tracking of the robotic platform, will be the method of choice in robotic surgery in the years to come. This is because they can be integrated into the robotic suite and thus require less external hardware.

With the proper registrations in place, surgical navigation has the potential to translate the wealth of preoperative information (e.g., multi-modal scans, target definition, most efficient route, critical organ segmentations) to the operating room, directly visualized in the surgical robot console. Using molecular imaging modalities (i.e., PET and SPECT), this has primarily been oncological data, providing guidance towards, e.g., (metastatic) lymph nodes defined before surgery [18].

Unfortunately, the use of preoperative images for surgical navigation is challenging in a soft tissue environment, where organs deform and where surgery itself changes the anatomy dramatically [69]. Efforts have been made to cope with these inaccuracies by regularly reapplying registration of the preoperative images to the current surgical situation using C-arms and ultrasound (e.g., [70, 71]), as well as tracked gamma probes [72] or gamma cameras [73].

Alternatively, to cope with soft tissue–induced deformation, it has also been proposed to navigate intraoperative molecular detection modalities, basically using them as pointers within the navigation workflow. In this way, navigation is useful for orientation and rough localization in the patient. Simultaneously, the real-time feedback of the molecular detection modality allows for correction and confirmation of the actual tissue targets once close enough. This concept has been shown in vivo using gamma probes (e.g., [74]) and fluorescence cameras [75–77], and preclinically even using robotic DROP-IN gamma probes [78] and robotic fluorescence cameras [33]. In our view, with the current status of this field, intraoperative imaging (e.g., ultrasound, freehand SPECT, fluorescence laparoscopy) remains indispensable in soft tissue environments.

### Advanced visualization and navigation strategies for preoperative molecular images

Before going to intraoperative molecular imaging approaches, it is relevant to discuss visualization techniques in more detail. To advance the integration between preoperative imaging and the surgical experience once the registration is solved, advanced medical image visualization strategies have been put forward.

#### Virtual reality

**Fig. 4:**
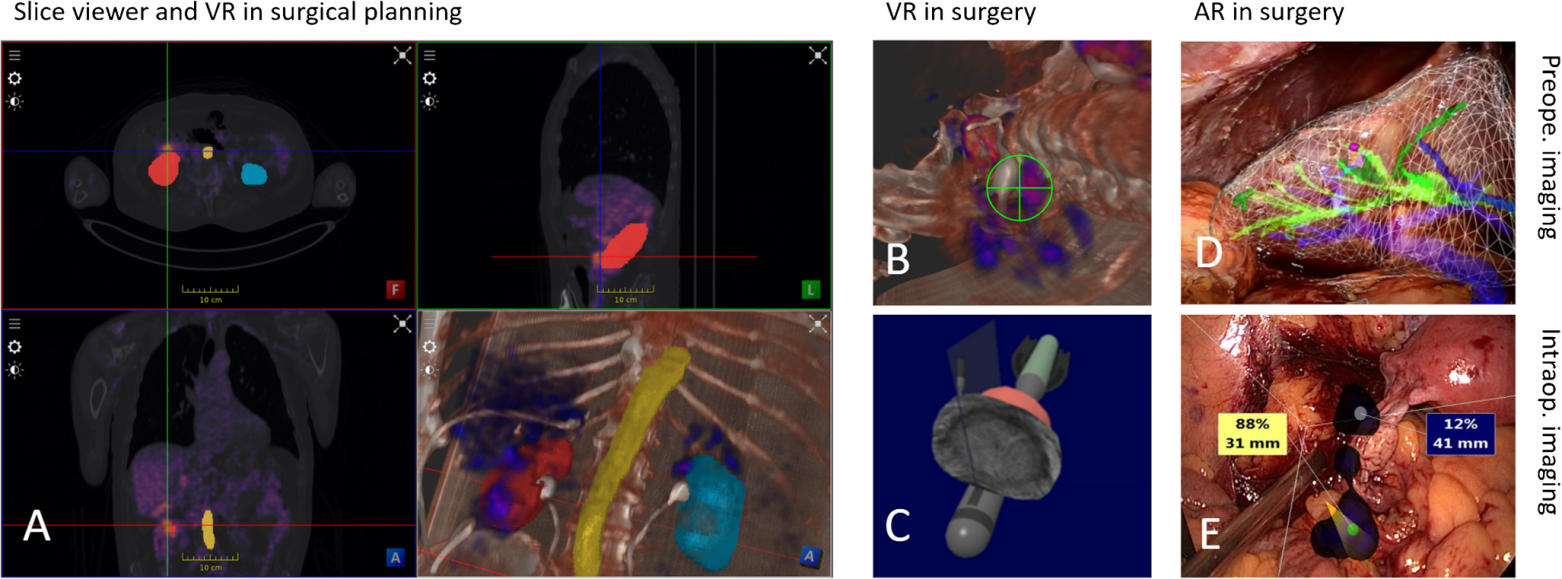
Different VR/AR visualization options applicable for robotic surgery. **A** Visualization of PET/CT in 3 planes (axial, coronal, and sagittal) and 3D render, including an overlay of segmented organs. **B** VR view of PET/CT image as used for guidance on PSMA-guided surgery. **C** VR view of intraoperative TRUS in the context of the TRUS probe and the robot instruments. Image courtesy of Tim Salcudean, UBC, Canada. **D** Segmented organs overlaid as AR patients’ body for port placement planning [80]. **E** AR visualization of freehand SPECT images showing sentinel lymph nodes in an endometrium cancer surgery

Since the 2000s, it has been possible to stream a digital full image viewer showing either 2D images or 3D images in three planes, often visualized as a 3D render in a virtual environment (i.e., virtual reality (VR); e.g., [79]) where a priori segmented organs and structures can be highlighted (Fig. 4A). This applies not only to preoperative images but also to intraoperative information, such as the read-out of intraoperative ultrasound (US), displayed in relation to the surgical instruments [67], giving the surgeon a virtual context during surgery (Fig. 4B).

#### Augmented reality

The next relevant improvement in this field came with the introduction of augmented reality (AR) into the operating room. Unlike the virtual environment provided with VR, AR is defined as an approach of visualization where “invisible” information (e.g., pre- or intraoperatively obtained imaging findings) are overlaid on the view of the real environment, providing immediate context with the actual patient (Fig. 4D and E). This concept was introduced in orthopedic and brain surgery as early as the 1990s [81] using CT/MRI and even in combination with microscopic imaging [82]. However, it took until 2000 for AR to make it to clinical applications [61].

**Fig. 5:**
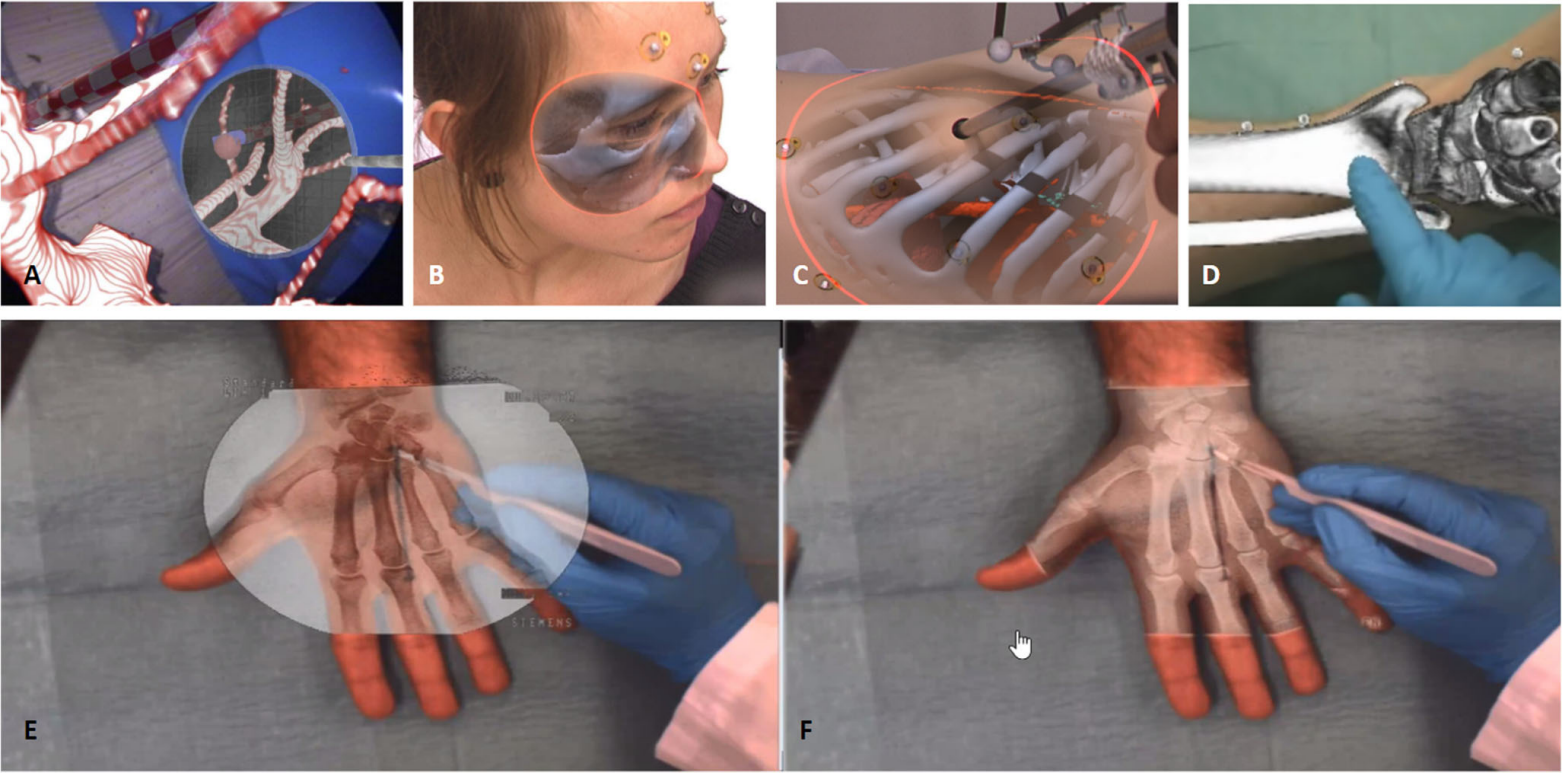
Depth perception improvement methods for AR: **A** virtual mirror, **B** curvature-dependent transparency, **C** virtual shadows, **D** object-subtraction; **E**, **F** example of a new paradigm of AR visualization where only relevant information is overlaid on the real image versus standard AR

A key challenge in AR is achieving a visualization that provides additional information without diminishing the camera’s information or giving a wrong perception. This limitation of AR has strongly limited its adoption. Different approaches have been proposed to improve depth perception (Fig. 5A–D), like the use of virtual mirrors [83], curvature-dependent transparency [84], virtual shadows [85], or object subtraction [86]. With the advances in machine learning, in particular, object subtraction has shown excellent performance for the detection of instruments in laparoscopy videos [87], opening a path towards visualization approaches where only the relevant information is overlaid to the surgeon [88] (Fig. 5E and F).

## Intraoperative planning, decision, and excision assistance

**Fig. 6:**
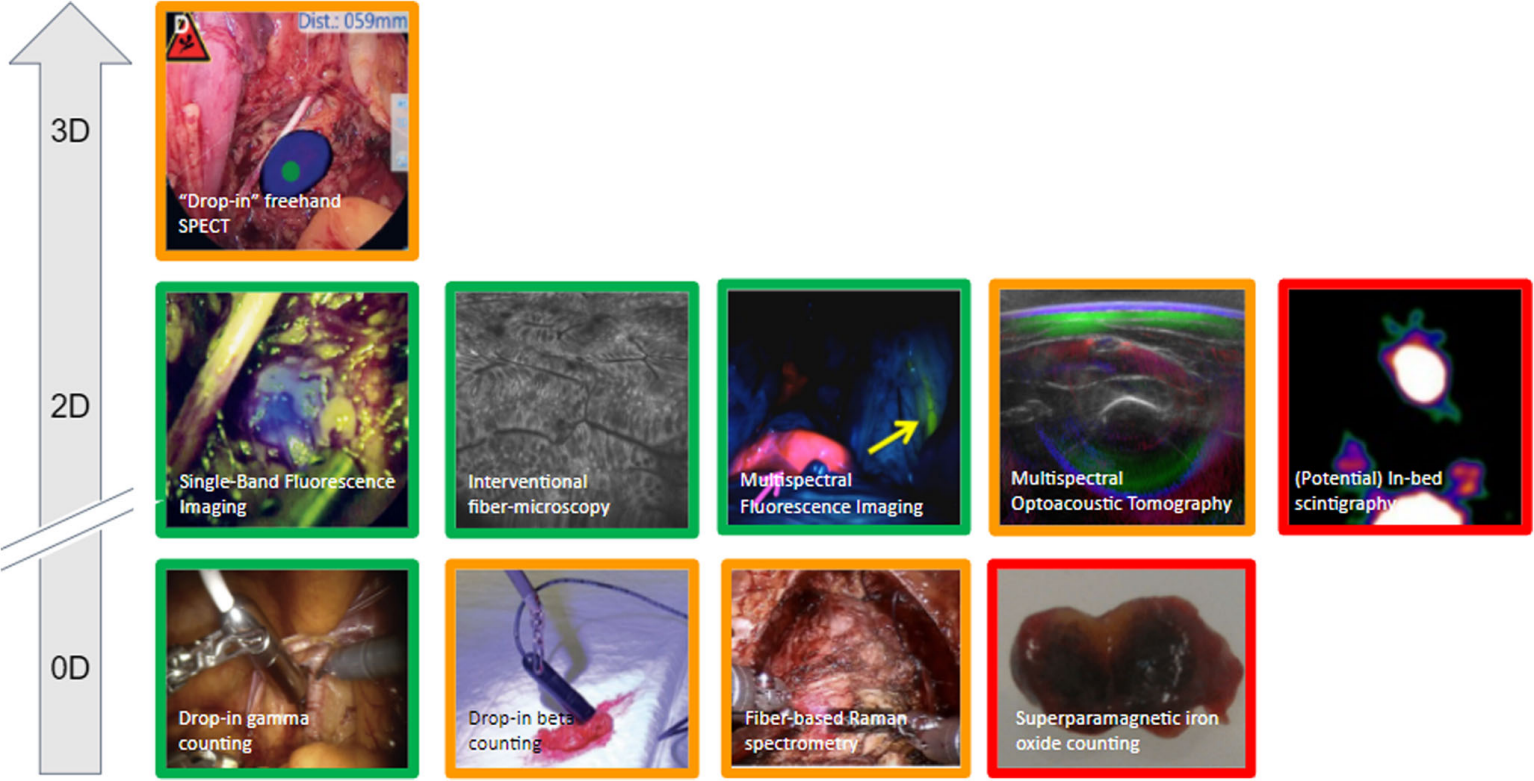
Non-exhaustive overview of some current and possible future technologies for robotic intraoperative molecular imaging separated by their dimensions and development status (green, commercially available; orange, research prototypes available; red, potential developments). Non-imaging devices are defined as zero-dimensional as they are a single pixel detector and not a line detector which would be one-dimensional

In addition to the planning and guidance delivered with preoperative scans, intraoperative imaging plays an important role in intraoperative lesion localization, decision making, and subsequent confirmation. Intraoperative imaging was first introduced in the context of X-rays at the beginning of the twentieth century and has since then evolved to provide a direct anatomical context within the operating room using, for example, a C-arm during orthopedic surgery [89]. Other forms of intraoperative anatomical imaging included the often used intraoperative US (e.g., to evaluate the extent of a tumor lesion during liver surgery [90]), or even intraoperative MRI (e.g., surgical management of glioblastoma [91]). However, molecular imaging has also played an essential role in image-guided surgery, especially using radio-guided surgery. Current intraoperative molecular imaging approaches mainly focus on radioactive, fluorescent, magnetic, or hybrid tracers, but also non-contrasted approaches are flourishing like multispectral optoacoustic tomography (MSOT; [92]), fiber-based microscopy [93], Raman spectrometry [94], among others (Fig. 6).

Before diving into the different intraoperative detection and imaging modalities, it is worth mentioning that navigation and advanced visualization concepts for preoperative images can be applied similarly using intraoperative molecular images. Techniques like freehand SPECT already included AR and pointer navigation means from its first publications [95–97]. The advantage of intraoperative imaging is that updated images, even after lesion removal, can be used to (partially) cope with tissue deformation during navigation [69]. Combined with real-time feedback from an intraoperative modality (e.g., radio- or fluorescence guidance) further confirms successful lesion localization [77].

### Radio-guided surgery

Being routinely applied for over several decades, radio-guided surgery is one of the most used types of (molecular) image-guided surgery [17, 18]. RGS focuses on the intraoperative detection and imaging of lesions or processes targeted with radiopharmaceuticals. The available detection modalities help realize in situ localization and provide an intraoperative control of successful resection. Being applied for a great amount of open and laparoscopic indications, the most used routine application has been the SLNB procedure (see Table 1). That said, many of the modalities developed to facilitate SLNB readily translate to, for example, receptor-targeted procedures. In receptor-targeted procedures, the main success story has been the radio-guided PSMA-targeted salvage surgery in prostate cancer, currently already applied in > 250 patients [98].

A relevant aspect of RGS is the fact that the patient is radioactive during surgery. This is less of a radiation protection issue for the patient: RGS is only performed if the patient’s benefits (i.e., removal of a tumor, minimally invasive lymphatic status, etc.) outweigh the potential risks (i.e., late radiation-induced cancer). However, it is for the surgical staff, who would else not receive any dose. This concern is interestingly less relevant in robotic surgery than in open or laparoscopic surgery, as fewer people stand around the patient. For procedures using low- to middle-energy gamma emitters (less than 250 keV; e.g., ^99*m*^Tc or ^111^In) or beta minus emitters (e.g., ^90^Y), the personnel’s radiation burden remains acceptable if all protective measures are taken [99]. However, this is not the case for RGS based on PET (e.g., ^18^F or ^68^Ga) or high-energy gamma emitters (e.g., ^131^I). In this case, beyond radioprotection means, it is recommended for surgeons and nurses to carry personal dosimeters and restrict the number of procedures per year depending on the isotope and the dose range used [100]. This downside of RGS has to be put in perspective with the wide variety of tracers available and the significantly higher penetration of gamma rays in tissue, allowing for in-depth detection possibilities.

Technologies that support the implementation of RGS concepts in the (robot-assisted) laparoscopic setting are discussed below.

#### Gamma counting

Gamma counting has been the most used surgical modality within RGS [17, 101]. Moving towards minimal-invasive procedures, long “laparoscopic” gamma probes are routinely used in sentinel lymph node biopsies (SLNB) in prostate cancer [102] and cervical cancer [103], but also in several other indications. Unfortunately, in the laparoscopic setting, their use is complicated by the limited movability. Being a rigid instrument, placement of laparoscopic gamma probes during tracing is restricted due to the limited range of motion available when working through a trocar [33]. This is further complicated in the robotic setting since the surgeon is no longer located in the sterile field. To improve positioning and at the same time regain autonomy for the surgeon and increase the range of motion available during tracing, a small-sized and tethered drop-in gamma probe was introduced [21, 33], a technology that is in line with the DROP-IN ultrasound technology that is used in robotic surgery [104]. Evaluations in prostate cancer SLNB indeed indicate an improved sentinel node detection rate for the DROP-IN (100%) versus laparoscopic gamma probe (76%) [22]. Recent studies indicate the DROP-IN also facilitates receptor-targeted surgery in the robotic setting (e.g., PSMA RGS [28]). These DROP-IN concepts applied to gamma probes in robotic surgery can be readily transferred to beta-probes [105] and as such allow for intraoperative detection of typical “PET-isotopes” (beta plus, e.g., ^18^F or ^68^Ga) and even opens the door for “therapeutic isotopes” (beta minus, e.g., ^90^Y).

#### Intraoperative scintigraphy

Portable gamma cameras can form a 2D image of the tracer distribution directly in the operating room [106–108] and probably even precede the use of gamma probes [109]. Initially developed for open surgery, their application has been shown in laparoscopic surgery as well, including prostate cancer, renal cell carcinoma, and testicular cancer SLNB procedures [110]. Although the gamma camera placement is more complicated during laparoscopy, its use helped verify the successful removal of the surgical targets. Providing only a 2D image, depth estimation is challenging, requiring frequent repositioning of the camera with different angles around the patient. To correlate the gamma image and the instruments in the field, radioactive markers on the instrument, e.g., a ^125^I-seed on the tip of them and a dual-isotope mode on the camera, have been proposed with improved usability [111]. We, ourselves, have tried to use such portable gamma cameras during robot-assisted surgery. Unfortunately, this proved complex, resulting in frequent collisions between the robot and the camera. Usage of such cameras during robotic surgery is most likely only valuable when directly integrated into one of the robotic arms, or possibly the patient bed [109]—a solution that unfortunately has not made it to product but is technically feasible. Alternatively, truly laparoscopic gamma camera prototypes have been evaluated in phantoms [112]. In a similar preclinical setting, other research groups have demonstrated a beta-cameras’ potential as a future application in (robot-assisted) laparoscopic RGS [18].

#### Freehand emission tomography

3D freehand imaging of radiopharmaceuticals is a method whereby a tracking system is used to determine the position and orientation of a nuclear detector (e.g., gamma probe or gamma camera) within the operating room, allowing for a 3D reconstruction based on the signal collected at these various positions [113]. The most established RGS form of this, and the only one being applied in vivo yet, is freehand SPECT [97]. Combining this technology with a tracked US device, intraoperative freehand SPECT/US has also made it to patients [114–117]. Preclinical research has also been performed to investigate freehand imaging of beta emissions [95, 118], high-energy gamma emissions [119], or even freehand PET imaging using a coincidence or time-of-flight principle with multiple detectors [120, 121]. While freehand SPECT is mainly used for open surgery procedures (e.g., SLNB or even receptor-targeted procedures [18]), this technology has also been translated to laparoscopic procedures, e.g., SLNB in gynecology and urology [77, 122] as well as radio-guided occult lesion localization in the lung [123]. However, the laparoscopic setting application is more challenging, where the limited movement of a laparoscopic probe restricts signal acquisition, and with that, reduces the quality of the freehand SPECT scan. Therefore, first steps are taken to translate the freehand imaging method towards robotic surgery using a tracked DROP-IN gamma probe [78, 124, 125]. Robotic SPECT has also been performed in a slightly different setting, using a robotic arm to autonomously create scans [126, 127]. These works have even extended to simultaneously acquire a cone-beam CT towards intraoperative SPECT/CT [128, 129]. Although the latter has not yet been tested in the surgical setting, one needs little imagination to hypothesize that such scans could be performed using robotic devices in the future. Interestingly, studies are being conducted to investigate if freehand SPECT can provide a reliable surgical roadmap (e.g., [130]). Some even suggest that freehand SPECT could potentially wholly replace the preoperative SPECT/CT. In line with this, no difference in performance was observed for 50 oral cancer SN procedures, where the surgeons were blinded for SPECT/CT [131].

### Fluorescence-guided and hybrid fluorescence-radio-guided surgery

Second to the application of RGS, fluorescence has seen a strong renewal of interest in the past decade [19, 132, 133]. Since an integrated laparoscopic camera is a crucial part of many robotic platforms, the interest in fluorescence imaging is even more emphasized by the fact that most surgical robots currently come with a laparoscope capable of fluorescence imaging.

#### Single-band fluorescence imaging

The most common fluorescence imaging type is single-band, meaning that it uses a single band-pass filter to depict one sole fluorescent “color.” In the clinic, fluorescence detection of the visible dye fluorescein and near-infrared (NIR) dye indocyanine green (ICG) is currently a routinely applied tool for angiographic purposes. An increasing number of trials are being conducted in the area of lymphatic mapping (e.g., [134]), perfusion-based tumor resection (e.g., [135]), and receptor-targeted imaging (e.g., [136]). The main advantage of fluorescence is that it provides real-time visual feedback concerning the tracer uptake during surgery, especially useful to confirm successful lesion localization during excision. Interestingly, while fluorescence images satisfy the surgeon’s demand for optical feedback, being based on light, it is inherently unsuited to visualize lesions located more than a couple of millimeters in tissue (i.e., < 1 cm depth [137]), something that is possible with for example nuclear medicine or radiological techniques. This was recently confirmed by Meershoek et al., demonstrating that in 52% of the patients, lesions were missed during robotic SLN procedures in prostate cancer if fluorescence imaging was used alone, underlining the need for additional technologies such as RGS [21]. This renders fluorescence imaging mostly useful for superficial applications and visual confirmation of successful target localization.

#### Multi-wavelength fluorescence imaging

Every fluorescent dye has its absorption and emission spectrum. By wisely choosing non-conflicting spectra of the individual dyes, several (targeted) fluorescent tracers can be depicted simultaneously using so-called multi-wavelength (also known as multispectral or multicolor) fluorescence imaging. First in-human studies illustrate this has the potential to separate different anatomical structures [138], a concept that could be especially interesting to improve the balance between surgically induced cure and side effects (e.g., decrease damage to healthy nerves and lymphatics [139, 140]). While most proof-of-concept studies have evaluated the use in microscopic neurosurgery (e.g., in glioblastoma [141]), there are also examples in laparoscopic surgery (e.g., parathyroid surgery [142], bladder cancer [143], liver cancer [144], and gynecology [145], or even robot-assisted laparoscopic surgery (i.e., prostate cancer [24])). Contrary to popular belief that only NIR-I (700–900 nm) is useful for fluorescence imaging, based on the relatively low tissue-induced absorption of light at these wavelengths, these studies do not visualize that much of a difference in intensity when used in a surgical environment, justifying the use of different wavelengths as well [146]. Even deeper NIR (e.g., 1000 to 1700 nm) has been suggested, theoretically providing higher resolution due to lower light scattering at these wavelengths [147]. However, limiting to the whole approach of multi-wavelength fluorescence is that only a small amount of fluorescent tracers is currently clinically approved (i.e., ICG, fluorescein, methylene blue, PpIX^5^-ALA/HAL [138]).

#### Hybrid imaging

Since detection techniques have their strengths and limitations, hybrid imaging (also referred to as dual-modal or bimodal imaging) combines the imaging properties of different modalities into a single technique, providing “best-of-both-worlds”. This is not only relevant for modalities that combine anatomical information with molecular information, but even for modalities that combine two types of molecular imaging, where these might provide guidance during different parts of the image-guided surgery process. This is the case for the currently trending topic of hybrid fluorescence-radio-guided surgery that combines both fluorescent and nuclear signatures into a single tracer [15, 148]. This concept was first introduced in the clinic in 2010 using ICG-99mTc-HSA nanocolloid [149] for SLNB in various forms of cancer (e.g., prostate [150], penile [151], head-and-neck [152], cervical [153], and breast cancer [154], as well as melanoma [155]). Currently, it has been applied in > 1500 patients, of which some were already treated in the robotic setting. Following the success in SLNB, this hybrid concept is now increasingly adopted for research in receptor-targeted tracers (e.g., neuroendocrine tumors using Cy5-^111^In-DTPA-Tyr3-octreotate [156], prostate cancer using PSMA I&F [157] and breast cancer using ^111^In-DTPA-trastuzumab IRDye800 (Wang2015)). There, some have recently even entered first-in-human studies (e.g., clear cell renal cell carcinoma using ^111^In-DOTA-girentuximab IRDye800 [30] or prostate cancer using ^64^Ga-PSMA-914 [29]).

### Other intraoperative molecular guidance methods

Given the penetration constraints of fluorescence and the fear patients, in particular, have concerning radioactivity, several research groups have turned to alternative physical events. In the following, we will describe three trending methods that are either already applicable to robotic surgery or where we foresee an application in robotic surgery shortly.

#### Superparamagnetic iron-oxide nanoparticle detection

Superparamagnetic iron-oxide nanoparticles (SPIONs) were introduced in 2013 as an alternative to radiolabelled colloids and blue dye in sLNB [158]. Clinically, their use has been restricted to SLNB (e.g., breast [159], vulva [160], prostate [161], penis cancer [162], and also melanoma [163]), or occult-lesion localization even in a laparoscopic setup [164]. As with radioactive tracers, SPIONs do allow for preoperative imaging and mapping of the sentinel nodes using MRI, where they usually show a black taint such that a degree of bare-eye guidance is possible. So far, the limitations in our view are in the lack of miniaturized detectors that can work in combination with surgical robots and slow tracer clearance from the body [165]. Interestingly, more and more groups are nevertheless actively working on bringing up tumor-specific SPIONs [166], which might offer alternatives for the future.

#### Multispectral optoacoustic tomography

The concept of using pulsed light in tissue while imaging the resulting ultrasonic vibrations to discern differences in tissue-absorption was introduced in 2007 for diagnosing breast cancer [167, 168]. Along with improvements of the technology itself, a significant breakthrough was achieved by applying fluorophores to increase tissue contrast in 2012 [169]. This technique, currently known as multispectral optoacoustic tomography (MSOT), has the main advantage of an improved tissue-penetration (up to 5 cm [92]) with comparison to fluorescence imaging and the fact that the resulting images are tomographic. Applications have been reported for lymphatic imaging [170], breast cancer detection [171], characterization of non-melanoma skin cancers [172], among others. Current research focuses on miniaturizing the devices to make them suitable for laparoscopic surgery and developing AI-powered signal processing algorithms to improve the interpretability generated images (e.g., [173, 174]).

#### Raman spectrometry

One of the promising technologies in the molecular imaging realm is Raman spectrometry, a technique that is based on the fact that different molecules, and with that different tissue types, have different Raman scattering spectra. This modality does not necessarily require a tracer. Its first applications in the operating room were as early as 2006 to evaluate resection margins in breast cancer [175]. Raman spectrometry can be applied to various indications like the detection of parathyroid adenoma [176] and the evaluation of lymph node metastasis in breast cancer [177], as well as the definition of resection margins in oral cancer [178, 179], glioma [180, 181], follicular thyroid cancer [182], and prostate cancer [94]. Given that Raman spectrometers can be easily miniaturized, porting them to a robotic setup is easy, as reported in the last reference. Like in MSOT, AI also has contributed significantly in particular by coping for illumination interference [183, 184] opening thus alternatives to generalize the use of this technology [185] and even beat fluorescence imaging in particular indications [186]. Using similar optical settings as with fluorescence imaging, tissue penetration is expected to be low (< 1 cm) making this a superficial technology.

## (Back table) Surgical verification

**Fig. 7:**
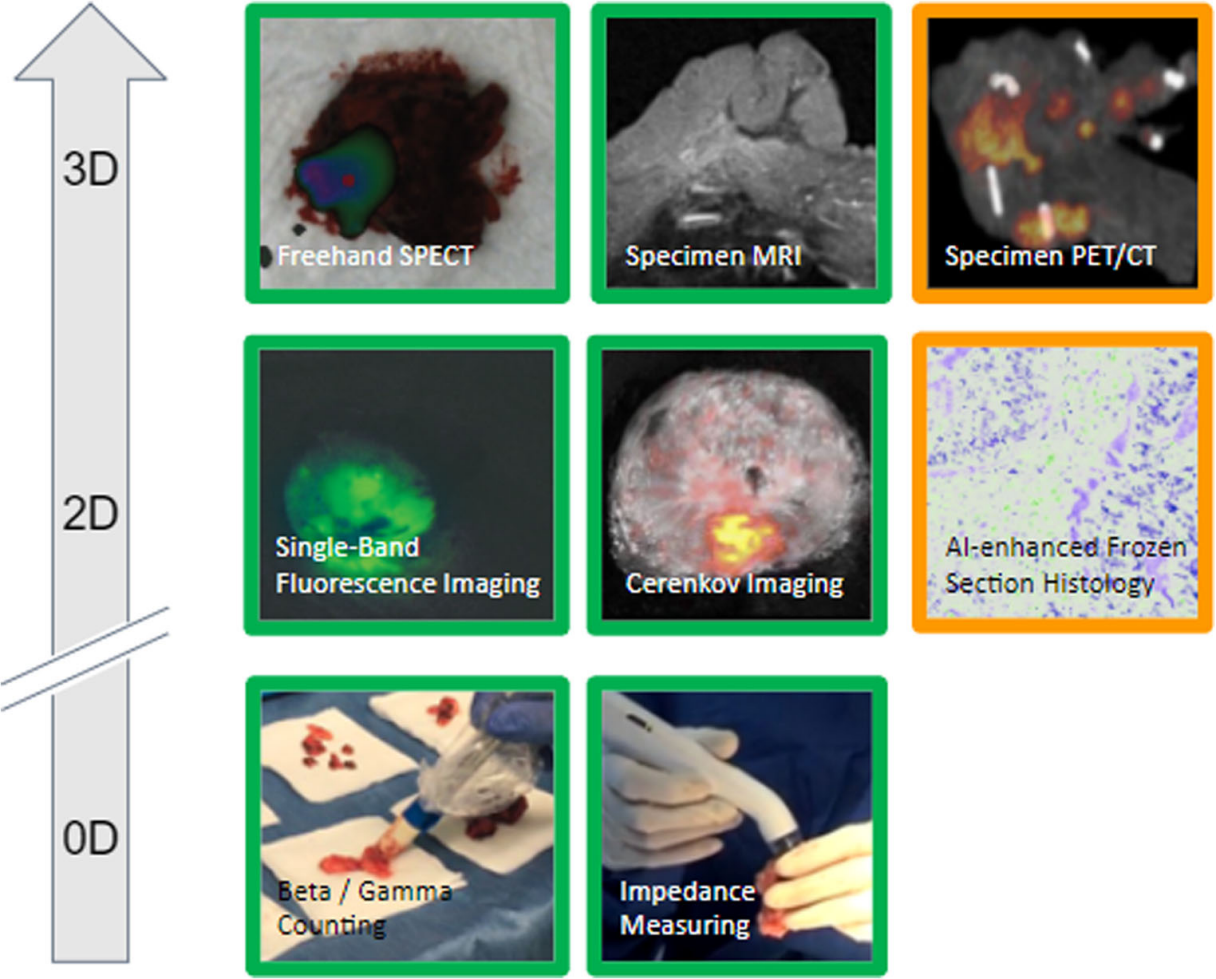
Non-exhaustive overview of some current and possible future technologies for back table specimen analysis/intraoperative pathological evaluation separated by dimensionality and development status (green, commercially available; orange, research prototypes available). Non-imaging devices are defined as zero-dimensional as they are a single pixel detector and not a line detector which would be one-dimensional

While preoperative and intraoperative molecular imaging have captured the spotlight over the last decades, the possibilities of ex vivo molecular imaging are not to be underestimated. Instrumental validation for all imaging procedures and the most reliable means to assess surgical resections’ accuracy still is pathology. Following surgery, patient reports always mention pathological outcome, based on which the clinical follow-up is being determined. In some cases, unfortunately, the pathological outcome may mean that patients have to be rescheduled for a second surgery. This inefficacy has driven the pursuit of intraoperative pathology and an additional role for ex vivo molecular imaging techniques to aid in the analysis of tumor margins and lymph node status (Fig. 7). Non-imaging and 2D imaging approaches play a significant role and will be discussed here. However, 3D methods like freehand SPECT [187, 188] or freehand fluorescence 3D surface imaging [189], or specimen PET/CT [190, 191] have been used for ex vivo imaging, providing valuable intraoperative information to the surgeon in terms of the necessity of margin extension or further surgical exploration.

### Intraoperative pathology

The introduction of frozen section histology has been a significant step towards high-speed pathological tissue analysis, which can be performed within the time frame of the surgery itself. Not only has this been widely applied during lymphatic mapping, where it is recommended in almost all indications besides breast cancer and melanoma [192], it is also routinely used in primary tumor resection wherever big margins are not possible to avoid functional or aesthetic side effects, e.g., in skin cancer [193], breast cancer [194], head and neck cancer [195], and prostate cancer [196, 197]. However, having a pathologist available for intraoperative assessments is a luxury most hospitals can not afford. The waiting time until results are reported must be minimized (extension of anesthesia, blocking of the operating room, lost time for OR personnel, etc.). As such, methods for faster surgical verification have been proposed. Here, AI has made its first steps into the field, providing preliminary evaluations or highlighting suspicious areas, thus supporting the pathologist’s efforts (e.g., [198, 199]). Such AI methods have great potential to accelerate the pathological process even further and might make intraoperative pathology logistically feasible for a lot more hospitals. One relevant alternative non-imaging approach is the intraoperative biochemical analyses of lymph nodes, like one-step nucleic acid amplification (OSNA). OSNA has made it to clinical routine in several countries and has shown diagnostic accuracy not only in breast cancer [200], but also in other tumor entities [201].

### Molecular non-imaging methods

Labeled tissue on the margins of specimens can be detected with non-imaging probes. This can be, for example, prostate tumors that are marked with PSMA [202] or sugar-avid tumors that are marked with FDG [203]. These methods can also verify if labeled structures were removed, like hyperactive parathyroids, or sentinel or metastatic lymph nodes. These control approaches are highly specific and are strongly recommended in guidelines for RGS. Alternatively, the tissue properties can be analyzed on-site using, for example, impedance/electromagnetic spectrometry [204], Raman spectrometry [186], or diffuse reflectance spectrometry [205], among others. Here, results are more controversial (e.g., [15]) and are not well spread in indications of robotic surgery.

### Molecular imaging methods

Of particular interest for robotic surgery are methods providing images, which can be played back in the surgeon’s console and thus, for example, letting him or her see which margins to extend. Intraoperative imaging modalities like portable gamma camera scintigraphy and single-band fluorescence imaging have been shown to provide valuable information not only in radio-guided occult lesion localization setups, for instance [206, 207], but also in more challenging applications like PSMA-guided surgery [29]. An interesting approach that has made it through the regulatory path into the market is Cerenkov luminesce. Where beta probes directly detect the emitted beta plus or beta minus particles of a radiopharmaceutical, it is also possible to detect the Cerenkov light with specialized, highly sensitive cameras [208–210]. Since most of this light is generated in the ultraviolet spectrum, the penetration depth is estimated at a couple of millimeters, similar to beta particle detection [211, 212]. In practical terms, specimens of patients given beta-emitters before surgery, such as 68Ga-PSMA or 18F-FDG, can be imaged in a light-tight device next to the OR table, providing an image in 1–5 min [213, 214]. This is needed as the amount of emitted luminescence is only in the range of a few photons per radioactive decay [215]. Non-tracer approaches are also available, being optical coherence tomography (OCT), one option that is picking up more and more momentum (e.g., [216]).

## Discussion and future perspectives

By connecting advances made in the field of medical devices (hardware and software) with tracer-based molecular imaging strategies, it becomes possible to provide a more comprehensive view of the direction that precision surgery is moving to. The same technologies are likely to disseminate to other surgical approaches and indications where robotic surgery is leading the way in this technical (r)evolution. One thing is clear, though: The future of precision surgery will rely on an interplay between pre- and intraoperative imaging, surgical hardware, and advanced visualization strategies enhanced by AI [34]. Based on the above-reviewed literature, below we summarize the trends and future perspectives that we have derived from the main steps relevant to molecular image-guided surgery.

For non-invasive (total-body) molecular imaging, nuclear medicine remains the golden standard with an extensive range of radiotracers available for various indications, mostly using either SPECT or PET imaging, combined with anatomical imaging like CT or MRI (e.g., [217, 218]). With the uprising of AI strategies, the first studies underline a great potential in molecular imaging to accelerate and optimize the process of disease diagnosis (e.g., [45]). But this is not where it stops. Since an AI algorithm has the potential to quickly compare current patient information with gigantic databases of previously treated patients (including patient scans, but importantly also metadata such as age, health state, comorbidities, blood values), such technology could also be used to suggest the most optimal treatment strategies (i.e., patient selection) and predict treatment outcome (e.g., [53]).

In the framework of surgical navigation based on preoperative imaging, AI also has the potential to assist in surgical planning by providing (semi-)automatic segmentation of the structures of importance [219]. Various technologies are already used to register these roadmaps to the patient in the operating room, but to not overly complicate logistics, we expect that in the robotic setting, these registration technologies will eventually converge to such which are directly integrated into the robotic platforms (e.g., laparoscopic video-based and US-based registrations) [67, 78]. However, due to patient deformation and repositioning, accurate registration of preoperatively acquired roadmaps remains challenging in soft-tissue anatomies. This will remain a big topic of research for the upcoming years. Nonetheless, since the robotic approach requires the surgeon to operate behind a (video) console, there is significant potential to integrate these navigated approaches using intuitive augmented reality visualizations directly (see Fig. 1B) that include AI for an optimized perception (see Fig. 5F) [88].

Intraoperative planning, decision-making, and assistance during the excision are perhaps the area that receives the most focus from the image-guided surgery point of view. In particular, the use of tracers to illuminate specific structures using radioactive or fluorescent signals is popular [69, 220]. Both strategies have clear advantages and disadvantages, which can be overcome using hybrid tracers. As these tracers provide both imaging signatures, they are compatible with different imaging modalities. Concerning the radioactive imaging signatures used, most radioactive detection is currently established around SPECT-based signals (i.e., low-to-mid–energy gamma emissions from isotopes such as ^99*m*^Tc and ^111^In) [221]. This still leaves a lot of room to investigate the value of alternative approaches using, for example, PET-based signals (i.e., beta plus or high-energy gamma emissions), beta minus emissions, or SPION detection, as well as alternative hybrid approaches with Cerenkov, multispectral/multiwavelength fluorescence, MSOT, and Raman spectrometry [16]. To make these imaging modalities, which often find their origin in open surgery, compatible with (laparoscopic) robotic surgery, we observe trends such as miniaturization, tethering, and positional tracking (e.g., [33, 78, 105, 222]). A likely future scenario is that more image-guidance modalities will eventually be wholly integrated into the robotic platform as done for fluorescence imaging. There, the robotic platform itself will provide high-accuracy positional tracking (i.e., vision- or US-based) to support navigation in pre- or intraoperative patient scans and allow for 3D freehand imaging.

Surgical verification is another area where molecular imaging is playing an emerging role. Imaging and counting modalities already enable confirming ex vivo the removal of structures seen both in preoperative images and detected intraoperatively (e.g., [202, 203, 207]). Dedicated specimen analysis methods will still play a role, in particular, there where intraoperative imaging cannot provide high image resolution ([208] vs. [119]). The more information becomes available and the better the registration, the more tasks will be taken over semi-autonomously by the robot [223]. From there, the step to step to autonomous robotic system is not far, and (intraoperative) image guidance will be instrumental. Several research groups have already shown that robots can already take actions based on previously defined trajectories derived from preoperative images (e.g., [224, 225]) or be guided by intraoperative imaging (e.g., [226]). More developments in that direction are to be expected.

## Conclusions

With the uprising of minimal-invasive robotic surgery, more and more instruments are becoming available to optimize surgical actions. Integration of molecular imaging might be the key to bringing precision surgery to the next level. The robotic platform seems to be ideal for the direct integration of image-guided surgery technologies. For most technologies, the way thereto is still long. Still, the research and engineering community and the exponential growth of AI are pushing towards precision surgery solutions that fully integrate preoperative, intraoperative, and postoperative imaging modalities to achieve an optimal patient outcome. Nuclear medicine plays a crucial role in this evolution, facilitating computer-assisted diagnosis, planning, (robotic) navigation and detection, and (back table) verification throughout the complete surgical route.

## Footnotes

1 https://www.ncbi.nlm.nih.gov

2 https://scholar.google.com/

3 https://www.semanticscholar.org/
